# Mechanical Properties and Cooperation Mechanism of Corroded Steel Plates Retrofitted by Laser Cladding Additive Manufacturing under Tension

**DOI:** 10.3390/ma17153690

**Published:** 2024-07-25

**Authors:** Lan Kang, Peng Song, Xinpei Liu, Haizhou Chen

**Affiliations:** 1 School of Civil Engineering and Transportation, South China University of Technology, Guangzhou 510641, China; 202221008814@mail.scut.edu.cn; 2 State Key Laboratory of Subtropical Building and Urban Science, South China University of Technology, Guangzhou 510641, China; 3 Centre for Infrastructure Engineering and Safety, School of Civil and Environmental Engineering, UNSW Sydney, Sydney, NSW 2052, Australia; xinpei.liu@unsw.edu.au; 4 China Construction Eighth Engineering Division Co., Ltd., Shanghai 200120, China; 202220107435@mail.scut.edu.cn

**Keywords:** laser cladding, additive manufacturing, mechanical properties, corroded steel plates, retrofit

## Abstract

As a metal additive manufacturing process, laser cladding (LC) is employed as a novel and beneficial repair technology for damaged steel structures. This study employed LC technology with 316 L stainless steel powder to repair locally corroded steel plates. The influences of interface slope and scanning pattern on the mechanical properties of repaired specimens were investigated through tensile tests and finite element analysis. By comparing the tensile properties of the repaired specimens with those of the intact and corroded specimens, the effectiveness of LC repair technology was assessed. An analysis of strain variations in the LC sheet and substrate during the load was carried out to obtain the cooperation mechanism between the LC sheet and substrate. The experimental results showed that the decrease in interface slope slightly improved the mechanical properties of repaired specimens. The repaired specimens have similar yield strength and ultimate strength to the intact specimens and better ductility as compared to the corroded specimen. The stress–strain curve of repaired specimens can be divided into four stages: elastic stage, substrate yield-LC sheet elastic stage, substrate hardening-LC sheet elastic stage, and plastic stage. These findings suggest that the LC technology with 316 L stainless steel powder is effective in repairing damaged steel plates in civil engineering structures and that an interface slope of 1:2.5 with the transverse scanning pattern is suitable for the repair process.

## 1. Introduction

During the service of metal structures, local damage such as corrosion and cracks often occur in structural element surfaces due to the effects of wear, environment erosion, and cyclic loads [1]. Such local damage leads to the loss of section thickness and local stress concentration, which decrease the bearing capacity of structural members and threaten the safety and durability of the structures [2,3]. Traditional repair technologies for the damaged steel members mainly include mechanical clamp [4], grout filling [5], adhesively bonded steel plates [6], or CFRP [7]. While these technologies can effectively enhance the stiffness and strength of damaged steel components, they may change the original morphology of the structure and require particular attention to the interface bonding condition [8]. Laser cladding (LC) is a technology in additive manufacturing in which injected metal powder is melted by a laser beam to form a clad, layer by layer, on the damaged surface of metal parts [9], with high efficiency, small heat affected zone (HAZ), and little distortion [10,11]. In recent years, the LC has been applied for the repair of damaged surfaces on various components such as railway wheels [12], moulds [13], turbine blades [14], and so on. And its application has gradually expanded to the repair of damaged steel structures. The LC technology can achieve a “restored repair” goal, in which the stiffness, strength, and geometric dimensions of the repaired member are nearly identical to those of the original member [15].

**Figure 1:**
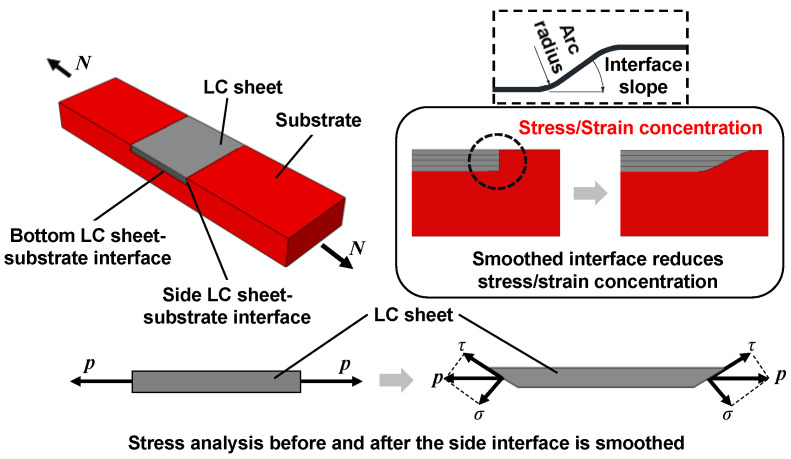
The mechanism of stress transfer between the LC sheet and substrate.

The cladding layer produced by LC can achieve a metallurgical bond with the substrate [16,17]. However, in the LC repair process, the interface quality is influenced by the LC process parameters and the interface geometry. Many research scholars have studied the influence of the LC process parameters on the microstructure and bond strength of the interface. Li et al. [18] studied the influence of laser powder on the interface characteristics and cracking behaviour of laser cladding-repaired nodular cast iron. Wang et al. [19] studied the influence of laser cladding process parameters and Al content on the microstructure and shear strength of the interface between Fe50Mn30Co10Cr10 and Al. Li et al. [20] employed Ni-Cu based alloy powders to repair invalid ductile cast iron components, investigating the influence of single-pass and multi-layer cladding processes on the microstructure and phase composition of the interface. Zhang et al. [21] studied the influence of laser power, scanning speed, and number of cladding layers on the interface layer between TA15 substrate and the TiAl claddings. However, few studies have focused on the effects of interface geometry on the final mechanical properties of repaired members. Cailloux et al. [22] studied the influence of trapezoidal groove geometry on the microstructure and mechanical properties of stainless steel 316 L parts repaired by laser cladding, and the results showed that a more frequent presence of pores at the interface as the opening angle decreases resulted in a decrease in mechanical properties. Kang et al. [23] employed 316 L stainless steel powder to repair locally corroded steel plates with different corrosion boundary treatments, and the tensile tests results showed that a smoothed corrosion boundary can reduce LC defects and prevent failure at the LC sheet–substrate interface. To further understand the results in reference [23], a stress analysis was conducted on the repaired member, and the mechanism of stress transfer between the LC sheet and substrate can be illustrated as shown in Figure 1.

As shown in Figure 1, when the interface slope is 90°, the stress transfers from the substrate to the bottom surface of the LC sheet through shear stress, while the stress transfers from the substrate to the side surface of the LC sheet through only normal stress, leading to a higher stress concentration and more LC defects. Therefore, the interface needs to be smoothed, modifying the stress transfer mechanism from only utilizing normal stress transfer to employing a mixed transfer of normal stress and shear stress, with the shear stress transfer being dominant, which is more reliable than normal stress transfer, thus reducing stress concentration. To achieve a good repair quality, it is necessary to determine the suitable interface slope, guided by experimental and numerical data.

The laser cladding layer offers high performance, including a dense microstructure, high microhardness, excellent wear resistance, and corrosion resistance [24,25,26], which is commonly used as a surface modification material [27]. However, when applying the LC technology to the field of civil engineering repair, the cladding layer transforms from merely a surface modification material into a load-bearing structural material. When the repaired member is subjected to external loads, the LC sheet is supposed to share the load with the substrate. Therefore, it is important to understand the mechanical properties of the LC sheet–substrate structure. In recent years, there have been studies on the mechanical properties of LC-repaired specimens. Zhang et al. [28] studied the mechanical performance of laser cladding-repaired bainite steel with AerMet100 steel under tensile tests, and the results showed that the overall mechanical properties of repaired specimens are comparable to the bainitic substrate. Chen et al. [29] used high-speed LMD technology to repair a 316 L austenitic stainless steel plate with a trapezoidal groove and analyzed the mechanical properties of repaired specimens under different laser energy densities. The results showed that the mechanical properties of the repaired specimens were superior to those of the rolled steel substrate at room temperature, and the specimens’ ultimate tensile strength and elongation at this temperature were positively correlated with the laser energy density. Rashid et al. [30] studied the effects of clad orientation on the tensile properties of 300 M cladded specimens, and the results showed that transverse cladding specimens with transverse clad orientation exhibited a higher tensile strength and ductility compared to the specimens with longitudinal and inclined clad orientation, and better tensile strength and Young’s modulus compared with grind-out only specimens. Zheng et al. [31] studied the effects of different heat inputs on the mechanical properties of 300 M steel repaired samples, and the results showed that the tensile strength of repaired samples with heat treatment increased by 75% and the elongation increased by 86% to 10.4% compared with the repaired samples without heat treatment. In summary, the current research mainly focuses on the overall mechanical properties and their influencing factors of repaired specimens, while studies on the strain distribution and cooperation mechanism of the LC sheet–substrate structure under load are limited.

The aim of this work is to obtain the cooperation mechanism of the LC sheet–substrate structure and determine the suitable interface slope and scanning pattern. Tensile tests and a finite element analysis were carried out to establish the influences of the interface slope and scanning pattern on the mechanical properties of repaired specimens. By comparing the tensile properties of the repaired specimens with those of the intact and corroded specimens, the effectiveness of the LC repair technology was evaluated. To investigate the cooperation mechanism between the LC sheet and substrate, a strain analysis of the LC sheet and substrate was carried out. This work will provide experimental guidance for repairing steel components with the LC technology.

## 2. Materials and Method

### 2.1. Materials

**Figure 2:**
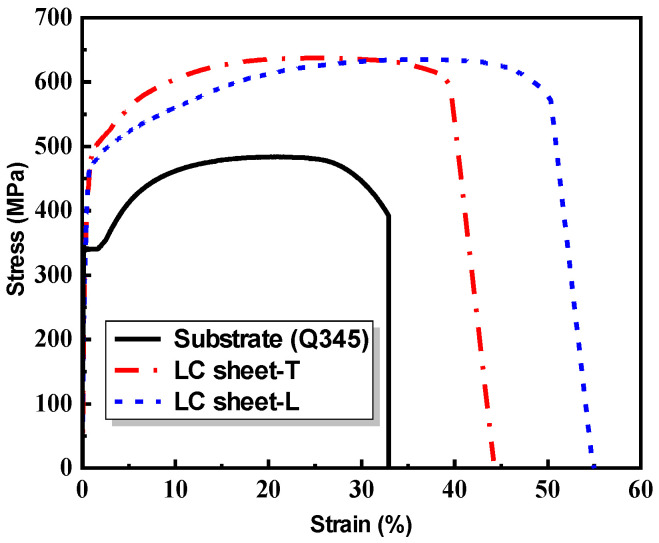
Stress–strain curves of the LC sheet and substrate.

The cladding material is 316 L stainless steel powder with a particle size of 53 to 150 μm, which was produced by the AVI Metal Additive Technology Co., Ltd. (Beijing, China). The chemical composition (in % by weight) of the 316 L stainless steel powder is presented in Table 1, according to the manufacturer’s datasheet. The substrate material is a Q345 steel plate with a thickness of 20 mm. Figure 2 shows the stress–strain curves of the LC sheets and substrate obtained from the flat coupon tension tests. Concerning the influence of laser scanning direction, the mechanical properties of the LC sheet with transverse scanning direction (named as LC sheet-T) and LC sheet with longitudinal scanning direction (named as LC sheet-L) are also presented. The mechanical properties of the substrate and LC sheet are summarized in Table 2. It can be noticed that the strength of LC sheet made of the 316 L stainless steel powder is greater than that of Q345 base metal plate.

### 2.2. LC Repair Process and Specimen Preparation

In this experiment, a commercial LC system consisting of a continuous wave fibre laser (MFSC-6000W, Maxphotonics Co., Ltd., Shenzhen, China), a six-axis mechanical arm (ABB Co., Ltd., Zurich, Switzerland), and a powder feeder system was used to control the cladding process. The process parameters are listed in Table 3.

**Figure 3:**
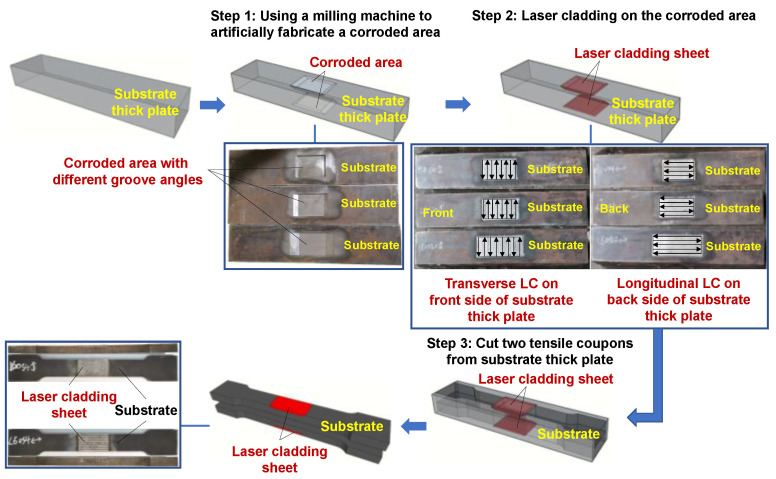
LC repair process.

**Figure 4:**
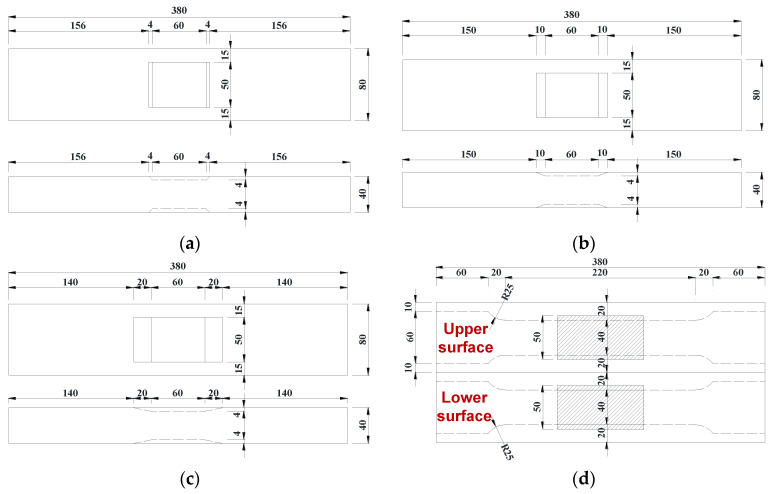
Dimensions of substrate thick plates (Unit: mm): (**a**) Trapezoidal groove with side slope of 1:1; (**b**) Trapezoidal groove with side slope of 1:2.5; (**c**) Trapezoidal groove with side slope of 1:5; (**d**) Dimensions of coupons.

The fabrication of repaired specimens involved three main steps as shown in Figure 3. Firstly, corroded area with a trapezoidal groove shape was artificially created on both the front side and back side of the substrate thick plate using a milling machine. Secondly, laser cladding with transverse and longitudinal scanning directions was performed on the front side and back side of the substrate thick plate, respectively, to form the LC sheet that filled the corroded area. Finally, two dog-bone specimens for tensile tests were cut from the substrate thick plate. In the first step, the corroded parts were directly removed by milling, disregarding the contribution of the corroded parts to the load-bearing capacity. This may lead to the results obtained from this experiment being lower in value than those under actual corrosion conditions. However, the greater the degree of localized corrosion, the lower the contribution of the corroded parts, and the artificial corrosion condition using milling process becomes closer to the actual corrosion condition. To investigate the influence of the interface slope, three types of grooves with different side slopes (1:1, 1:2.5, and 1:5) were machined on three substrate thick plates in the first step, respectively, as shown in Figure 4, to fabricate the repaired specimens.

**Figure 5:**
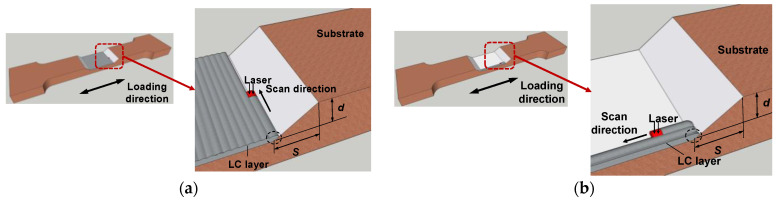
(**a**) Transverse LC; (**b**) longitudinal LC.

**Figure 6:**
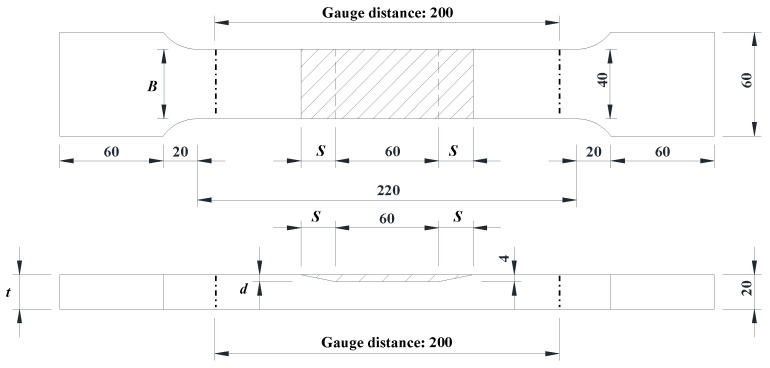
Designed dimensions of specimens (unit: mm).

Figure 5 illustrates the detail of the laser scanning pattern. In the transverse scanning pattern, the scanning directions of all layers are the same and perpendicular to the loading direction. In the longitudinal scanning pattern, the scanning directions of all layers are the same and parallel to the loading direction. The designed dimensions of the specimens are shown in Figure 6.

In this experiment, three groups of repaired specimens were fabricated with an interface slope (*S*/*d*) of 1:1, 1:2.5, and 1:5, as listed in Table 4. Each group contains two specimens, one with the transverse scanning pattern and the other with the longitudinal scanning pattern, as shown in Table 4. To evaluate the effectiveness of the repaired specimens, three intact specimens and one corroded specimen were prepared for comparison, which precede the nomenclature with -Int and -Cor, respectively.

**Figure 7:**
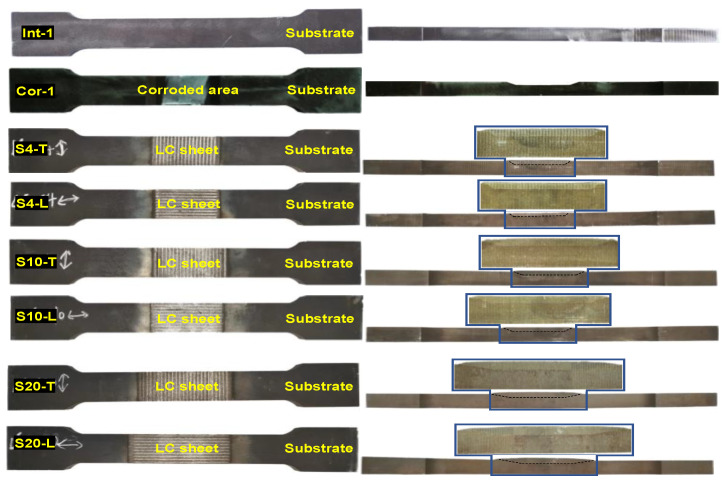
Photographs of specimens.

**Figure 8:**
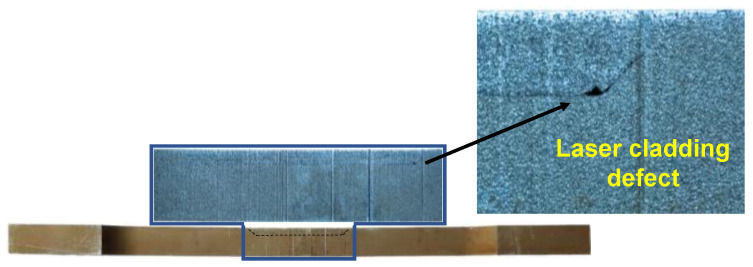
LC defect in specimen with interface slope of 1:1.

The parameters *B*, *t*, *d*, and *S* are the width, thickness, corrosion depth, and corrosion slope length of specimens, respectively. The parameter *t*_LC_ is the thickness of the LC sheet. The subscripts _des_ and _mea_ mean that the variable belongs to the designed and measured value. The nomenclature for the repaired steel plate specimens followed the format of S××−•, in which S×× represents the corrosion slope length, and • represents the coupon’s scanning pattern. For instance, Specimen S4-T indicates a specimen produced using the scanning pattern T, with a 4 mm corrosion slope length. Figure 7 shows the photograph of the prepared specimens. It can be observed that the geometric dimensions of the repaired specimens are almost identical to those of the intact specimens. Additionally, pores were found at the corner of the interface in the specimen with interface slope of 1:1, as shown in Figure 8. This is due to the lack of fusion defect caused by the poor accessibility of the projection nozzle during the LC process, which becomes more pronounced as the interface slope increases.

### 2.3. Experimental Setup

**Figure 9:**
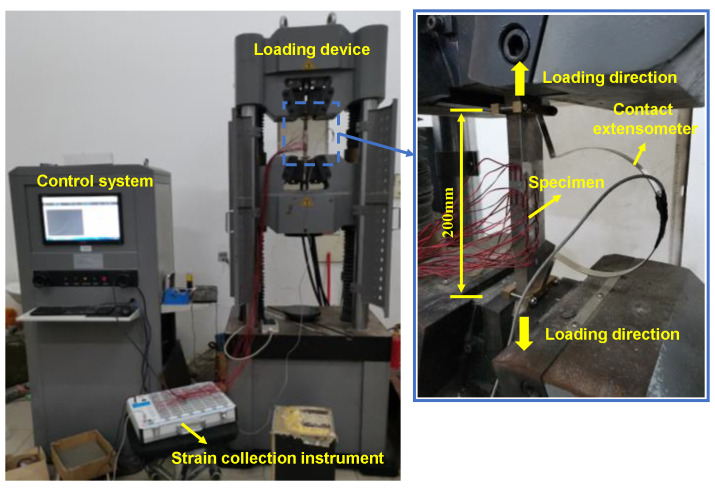
Test setup.

**Figure 10:**
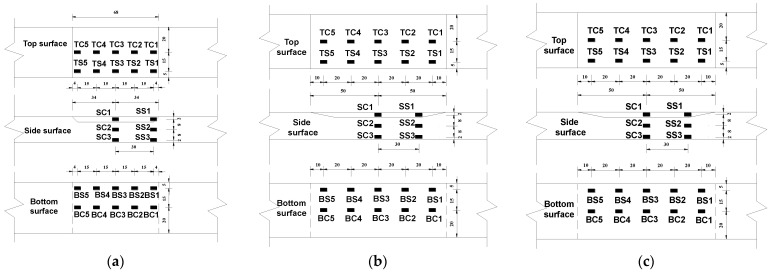
Gauge arrangement (unit: mm): (**a**) S4 specimens; (**b**) S10 specimens; (**c**) S20 specimens.

A 1000 kN electro-hydraulic servo universal testing machine consisting of loading device and control system (Material Testing, Changchun, China) was used for tensile tests on the specimens. The specimen was clamped by wedge grips at both ends. The displacement of the specimens was measured using a contact extensometer with a gauge length of 200 mm and a maximum measured deformation of ±50 mm, as illustrated in Figure 9. To obtain the strain distribution and variations in the repaired specimens during loading, a total of 26 strain gauges were arranged on the top (LC sheet), side, and bottom surfaces of the repaired specimens, as shown in Figure 10. The strain data were collected using the multi-functional static strain testing system (JM3813) in combination with the static testing software. According to the Chinese standard GB/T 228.1-2010 [32], the loading procedure was displacement-controlled with a loading rate of 0.2 mm/min before the peak load and 0.5 mm/min after the peak load, with a gradual transition between the two rates.

## 3. Results and Discussion

### 3.1. Failure Mode

**Figure 11:**
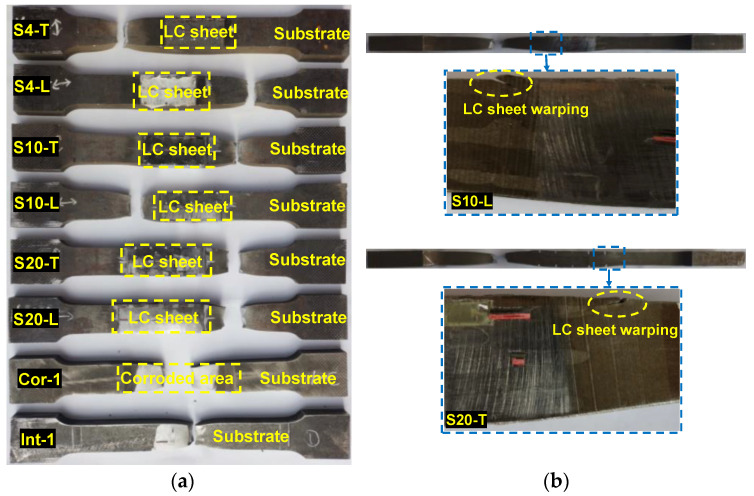
(**a**) Failure modes of specimens; (**b**) warping at the LC sheet edge.

The failure modes of the test specimens are depicted in Figure 11, which shows that the corroded specimen fractured at the middle section was weakened by the corrosion. However, all the repaired specimens fractured at the pure substrate section, which is relatively weaker after the corroded section was repaired and reinforced with the cladding material using the LC technology. Therefore, it can be concluded that the corroded steel plates repaired by LC technology can reinforce the weakened section caused by the local corrosion, preventing section failure at the locally corroded area. Notably, the failure modes of the repaired specimens were unaffected by the interface slope and scanning pattern. Nevertheless, it was observed that local warping and fracture occurred at the LC sheet edge, as indicated in Figure 11b, suggesting that stress concentration was present during the loading procedure. To address this issue, it is recommended to perform laser remelting on the outer boundary of the LC sheet to enhance the bonding between the LC sheet and substrate, thus reducing the stress concentration [33].

### 3.2. Mechanical Properties

**Figure 12:**
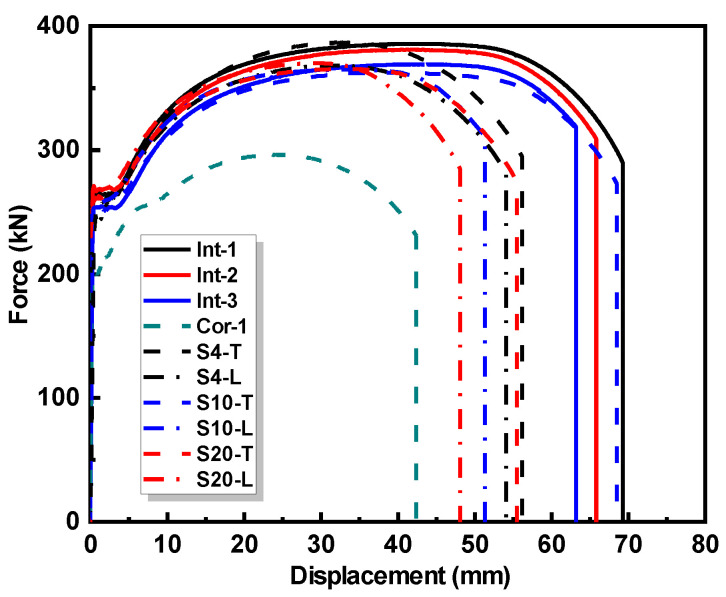
Force–displacement curves of specimens.

The force–displacement curves of the specimens obtained form the tensile tests are presented in Figure 12.

It can be observed that the presence of the corrosion area significantly degrades the force–displacement curve of the corroded specimen Cor-1. Compared with the intact specimens, the corroded specimen exhibited a shorter yield plateau and a significantly lower yield load, ultimate load, and ultimate displacement. The repaired specimens exhibited a similar yield load and ultimate load, albeit with a lower ultimate displacement, compared with the intact specimens.

**Figure 13:**
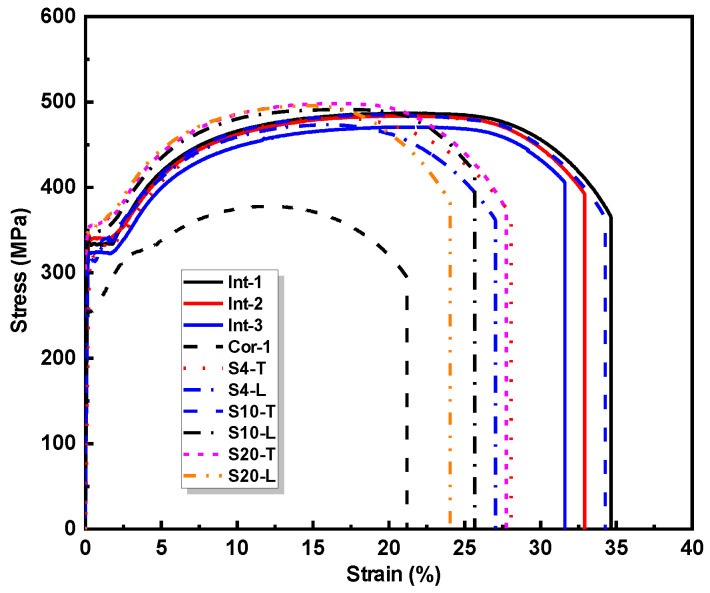
Stress–strain curves of specimens.

To mitigate the influence of varying specimen sizes on the test results, the stress and strain at different loading moments were calculated based on the cross-section area of the specimens. Since the failure of all the repaired specimens occurred in the pure substrate section, the cross-section area for calculation is taken from the pure substrate section. Figure 13 illustrates the full range stress–strain curves of the test specimens.

To compare the mechanical properties of different specimens, Table 5 lists the mechanical performance indicators such as the elastic modulus, yield strength, ultimate strength, and elongation.

Additionally, to assess the effectiveness of the LC repair technology, we introduced parameters *I*_y_ and *I*_u_ in Table 5, which quantify the improvements in yield strength and ultimate strength, respectively, of both intact and repaired specimens when compared to the corroded specimen. The expressions for calculating *I*_y_ and *I*_u_ are provided below:

(1) $$I_{y}=\frac{\sigma_{y,\mathrm{Compared}}-\sigma_{y,\mathrm{Corroded}}}{\sigma_{y,\mathrm{Corroded}}}\times 100\%$$

(2) $$I_{u}=\frac{\sigma_{u,\mathrm{Compared}}-\sigma_{u,\mathrm{Corroded}}}{\sigma_{u,\mathrm{Corroded}}}\times 100\%$$

where *σ*_y,Compared_ is the yield strength of the repaired or intact specimens; *σ*_y,Corroded_ is the yield strength of the corroded specimen; *σ*_u,Compared_ is the ultimate strength of the repaired or intact specimens; and *σ*_u,Corroded_ is the ultimate strength of the corroded specimen.

As shown in Table 5, the average elastic modulus of the intact specimens was 197.66 GPa, which was close to that of the corroded specimen, namely, 200.19 GPa. This is due to the use of the same substrate material of the intact and corroded specimens. However, the elastic modulus of the repaired specimen was determined by the weighted average of the elastic modulus of the substrate material and cladding material. Since the elastic modulus of the cladding material was lower than that of the substrate material, the elastic modulus of the repaired specimens was lower than that of the intact and corroded specimens, as observed in Table 5. It can be seen that the elastic modulus of the repaired specimens increases as the interface slope decreases. It can be observed from Table 5 that the yield strength and ultimate strength of the intact specimens were significantly higher than those of the corroded specimen. Similarly, the yield strength and ultimate strength of the repaired specimens were significantly higher than those of the corroded specimen. By comparing the *I*_y_ and *I*_u_ of the intact and repaired specimens, it can be observed that the improvement in yield strength and ultimate strength of the repaired specimens is close to that of the intact specimens when compared to the corroded specimen. Furthermore, the average ultimate elongation of the repaired specimens was 27.81%, which was higher than that of the corroded specimen (21.20%), but lower than that of the intact specimens (33.05% on average). The results suggest that the repaired specimens have a similar yield strength and ultimate strength to the intact specimens and better ductility as compared to the corroded specimen.

By comparing the mechanical properties of the repaired specimens with different interface slopes, as shown in Table 5, it can be found that the yield strength and ultimate strength slightly improved as the interface slope decreased. This may be due to the higher material strength of the LC sheet than that of the substrate. As the interface slope decreases, the proportion of the LC sheet increases, resulting in better local reinforcement of the repaired specimens. Meanwhile, the decrease in the interface slope comes at the expense of more cladding material consumption in the LC repair process. It can be observed that the interface slope has no impact on the ultimate elongation of the repaired specimens. The scanning pattern has no impact on the yield strength and ultimate strength of the repaired specimens. The ultimate elongation of the repaired specimens using the transverse scanning pattern was larger than that of the repaired specimens using the longitudinal scanning pattern, as shown in Table 5.

### 3.3. Cooperation Mechanism between LC Sheet and Substrate

**Figure 14:**
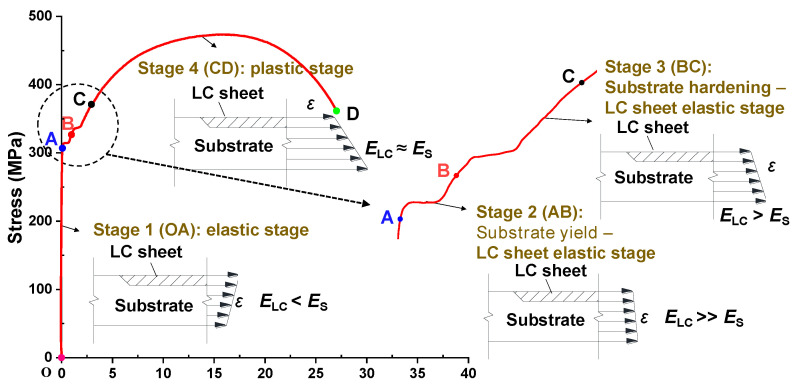
Cooperation mechanism between LC sheet and substrate.

It can be observed from Figure 13 that the stress–strain curves of some repaired specimens exhibit a distinct ‘two-plateau’ characteristic. Based on the stress–strain relationship of the substrate and LC sheet as shown in Figure 2, it can be observed that the substrate yields earlier than the LC sheet. Therefore, the typical stress–strain curve of the repaired specimen can be divided into four stages: elastic stage (OA), substrate yield-LC sheet elastic stage (AB), substrate hardening-LC sheet elastic stage (BC), and plastic stage (CD), as shown in Figure 14, which was plotted based on the test results of specimen S4-L.

The four stages illustrate the cooperation mechanism between the LC sheet and substrate, which can be explained as follows. In the elastic stage, the elastic modulus of the LC sheet was lower than that of the substrate, as indicated in Table 2. Based on the equal stress condition of laminated composite materials [34], the strain in the LC sheet is larger than that in the substrate. In the substrate yield-LC sheet elastic stage, the strain in the substrate increases faster than that in the LC sheet, making the strain in the substrate gradually exceed that in the LC sheet. In the substrate hardening-LC sheet elastic stage, the strain rate of the substrate increases, while the strain rate of the LC sheet remains constant, making the strain in the substrate further exceed that of the LC sheet. Finally, in the plastic stage, both the substrate and LC sheet are in plastic deformation, and their strain rates become comparable, and thus, the strain in the substrate remains larger than that in the LC sheet.

**Figure 15:**
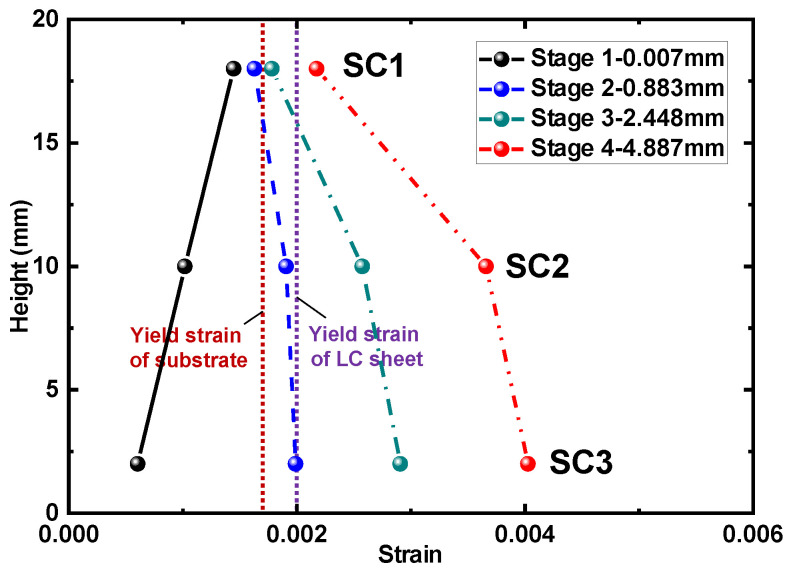
Strain distribution of side surface of specimen S4-L in four stages.

To verify the above discussion, the strains measured by strain gauges during the experiment were analyzed. Figure 15 shows the strain distribution across the section thickness of specimen S4-L in the four stages, which is consistent with the strain distribution in the four stages discussed above. Specifically, during the elastic stage, the strain of the substrate was lower than that of the LC sheet, but it gradually increased and became larger than that of the LC sheet as the load was applied.

**Figure 16:**
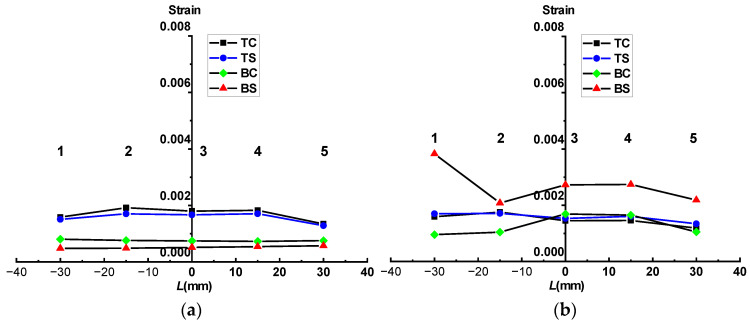

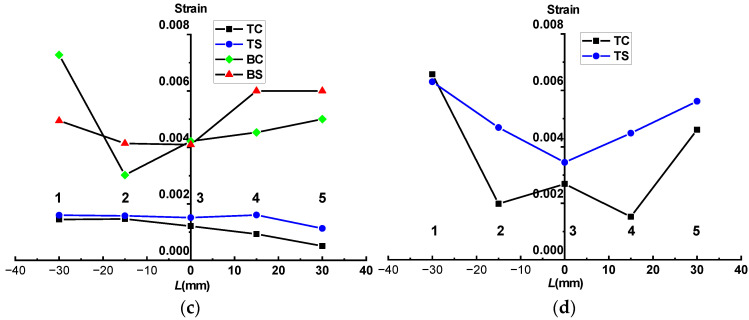
Strain distribution of top and bottom surfaces of specimen S4-L in four stages: (**a**) Stage 1: 0.007 mm; (**b**) Stage 2: 0.883 mm; (**c**) Stage 3: 2.448 mm; (**d**) Stage 4: 4.887 mm.

Figure 16 illustrates the strain distributions at the top and bottom surfaces of specimen S4-L in the four stages.

It can be seen that in stage 1, the strain at the LC sheet (top surface) was higher than that at the substrate (bottom surface), as shown in Figure 16a. However, as the load was continuously applied, the strain in the substrate increased rapidly and gradually surpassed that in the LC sheet in stage 2 and stage 3, as demonstrated in Figure 16b,c. In stage 4, due to the excessive strain at the bottom surface, which was beyond the capacity of the strain gauges to measure, Figure 16d only shows the strain distribution at the top surface. As depicted in Figure 16d, the strain at the edges of the LC sheet was significantly greater than that at other locations, indicating that the stress was concentrated at the LC sheet edges, leading to local warping, as observed in Figure 11.

### 3.4. Finite Element Analysis

**Figure 17:**
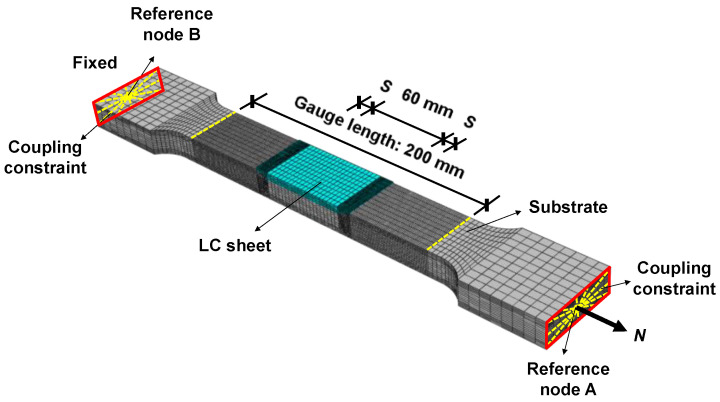
FE model.

To verify the experimental results, a finite element (FE) analysis was carried out. The FE model for simulating the tensile behaviour of the test specimens was developed using the ABAQUS software package (2021). The 3D solid reduced integration element C3D8R was chosen as the element type for the whole FE model. To ensure the computational efficiency and accuracy of the FE model, different mesh sizes were adopted for different regions in the model according to the distribution of stress and strain. As shown in Figure 17, the mesh in the pure substrate section region where the fracture may occur was refined with a size of 1 mm, and the region where the LC sheet was located was meshed with a mesh size of 3 mm except for the side boundaries of the LC sheet, which were meshed with a mesh size of 0.5 mm.

The shape and size of the mesh were set symmetrically in the thickness and length directions of the specimens. In the FE model, the fixed end surface was coupled to reference node B, while the loading end surface was coupled to reference node A. The FE model for each specimen was built based on the measured dimensions as listed in Table 4. The interface between the LC sheet and the substrate was assumed to be fully bonded [17]. The effects of the residual stress generated by the LC process were not considered in the FE model.

**Figure 18:**
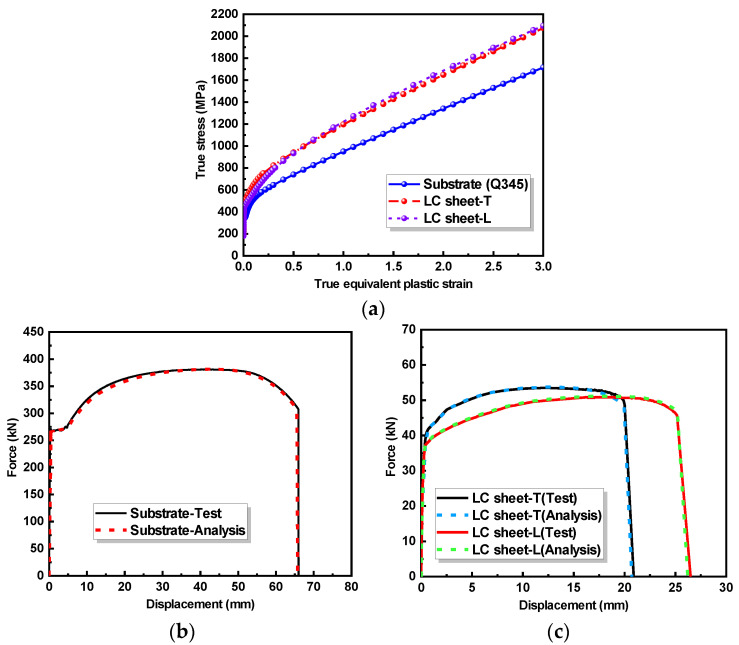
Material constitutive relationships: (**a**) constitutive relationship curves of substrate and LC sheets; (**b**) force–displacements of substrate; (**c**) force–displacements of LC sheets.

The material constitutive relationships for the substrate and LC sheets with scanning patterns T and L are shown in Figure 18.

By applying the average weight whole-process methodology [35,36], the engineering stress–engineering strain curves of the substrate and LC sheets, as given in Figure 2, were converted into true stress-equivalent plastic strain curves as required by ABAQUS as shown in Figure 18a, and the weight parameter Q was obtained as shown in Table 2. To simulate the post-peak phase of the material’s stress–strain curve, the ductile fracture model from the literature [35,36] was also adopted in the FE analysis and the toughness parameter α was obtained as shown in Table 2. Figure 18b,c show that the numerical results using the material constitutive relationships in this section agree well with the coupon test results.

**Figure 19:**
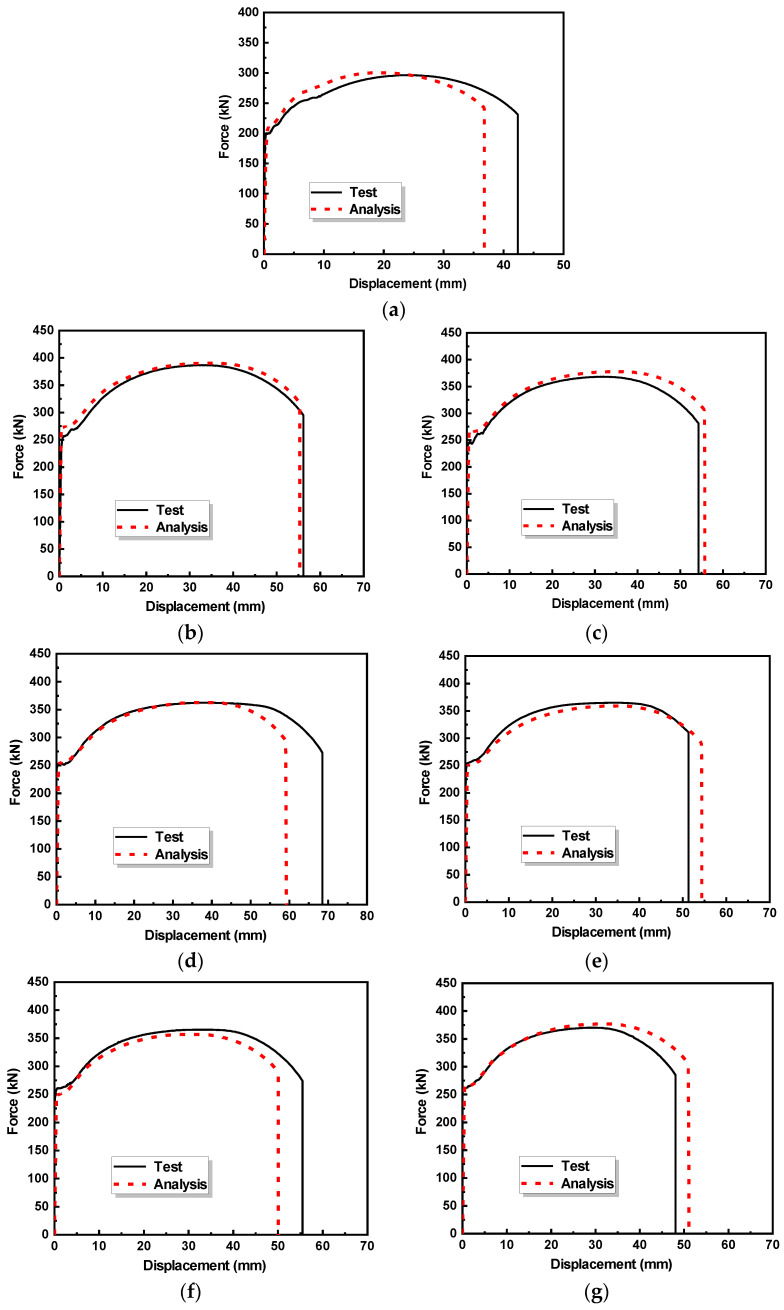
Comparisons between experimental and numerical force–displacement curve: (**a**) Cor-1; (**b**) S4-T; (**c**) S4-L; (**d**) S10-T; (**e**) S10-L; (**f**) S20-T; (**g**) S20-L.

By comparing the FE analysis results with the experimental results, the developed FE model and experimental results were mutually validated. Figure 19 shows comparisons of the force–displacement curves obtained from both the FE analysis and experimental tests.

Good agreement between the numerical and experimental results is observed, and the stiffness, yield load, and ultimate load of the numerical and experimental results are very close. Therefore, the developed FE model is capable of predicting the tensile behaviour of corroded steel plates repaired by the LC technology.

**Figure 20:**
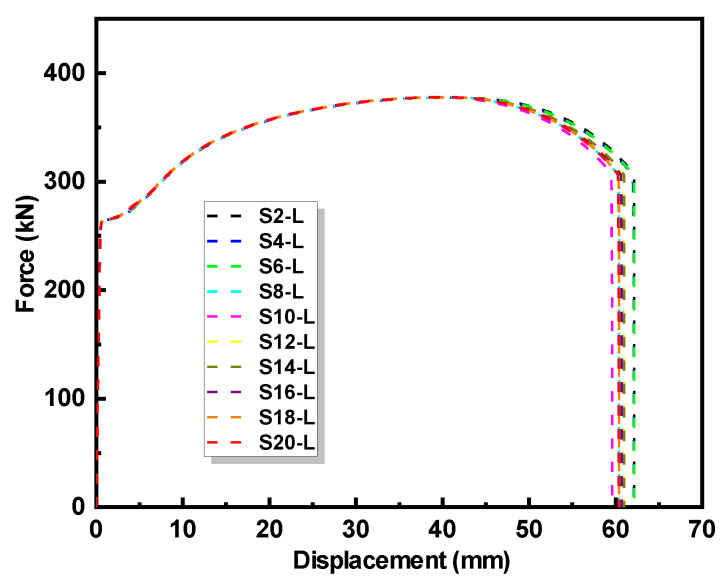
Comparisons of force–displacement curves with different interface slopes.

Upon validation of the developed FE model, a parameter analysis was carried out to further explore the effects of the interface slope on the mechanical properties of the repaired steel plates. The scanning pattern L was employed for the LC sheet of all models. The parameter analysis involved ten repaired steel plate models with identical geometric dimensions and a corrosion depth of 4 mm, only varying the corrosion slope length of 2, 4, 6, 8, 10, 12, 14, 16, 18, and 20 mm, which led to values of the interface slope *d*/*S* ranging from 1:0.5 to 1:5. Figure 20 depicts the force–displacement curves with the different interface slopes obtained from this parameter analysis.

The results reveal a slight decrease in the ultimate elongation of the repaired steel plates as the interface slope decreases, while the initial stiffness, yield strength, and ultimate strength remain relatively unchanged. This finding shows a minor deviation from the experimental observations, which can be explained by the variations in the thickness of the LC sheet and the influence of the LC defects in the experiment. Nevertheless, both the numerical and experimental results indicate that the interface slope has a minimal impact on the mechanical properties of the repaired steel plates.

## 4. Conclusions

The effects of the interface slope and scanning pattern on the mechanical properties of corroded steel plates repaired by the LC technology and on the cooperation mechanism between the LC sheet and substrate have been experimentally investigated. The effectiveness of the LC technology for the repair of corroded steel plates was confirmed by a finite element analysis. The conclusions can be summarized as follows:

1. The corroded steel plates repaired by the LC technology can prevent failure at the locally corroded area. The repaired specimens have a similar yield strength and ultimate strength to the intact specimens and better ductility as compared to the corroded specimen, while the geometric dimensions are almost identical to those of the original state.
2. A larger interface slope between the substrate and LC sheet is more likely to cause a lack of fusion defects during the LC process. The yield strength and ultimate strength of the repaired specimens slightly increase as the interface slope decreases, yet this comes at the expense of more cladding material consumption in the LC repair process. The LC scanning pattern has no impact on the yield strength and ultimate strength of the repaired specimens, while the ultimate elongation of the repaired specimens using the transverse scanning pattern is larger than that of the repaired specimens using the longitudinal scanning pattern. The interface slope of 1:2.5 and transverse LC scanning pattern were suitable for the repair of corroded steel plates.
3. The typical stress–strain curve of repaired specimens can be divided into four stages: elastic stage, substrate yield-LC sheet elastic stage, substrate hardening-LC sheet elastic stage, and plastic stage. During the elastic stage, the strain of the substrate was lower than that of the LC sheet, but it gradually increased and became larger than that of the LC sheet as the load was applied.
4. The stress concentration at the LC sheet edges led to local warping at the LC sheet edges when the repaired specimen failed under tension.

These findings suggest that the LC technology is effective for the repair of corroded steel plates in civil engineering structures. In practical projects, the corroded area can be pre-treated to form an appropriate interface slope to achieve good repair quality. The performance difference between the cladding material and substrate material can influence their cooperation mechanism. Future research will explore the mechanical behaviour of repaired steel components using various cladding and substrate materials, as well as their behaviour under different loading conditions such as bending.

## Figures and Tables

**Table 1:** Chemical composition (in % by weight) of 316 L stainless steel powder.

Chemical Composition	Si	Cr	Ni	Mn	Mo	C	O	Fe
in % by weight	0.59	17.3	12.23	1.22	2.17	0.013	0.029	Balance

**Table 2:** Material properties.

Materials	*E* (GPa)	*μ*	*f*_y_/*σ*_0.2_ (MPa)	*σ*_u_ (MPa)	*σ*_neck_ (MPa)	*ε*_neck_	*Q*	*α*
Substrate	197.7	0.30	332.1	480.4	578.9	0.1838	0.40	2.10
LC sheet-T	178.0	0.29	383.2	607.6	773.5	0.2414	0.47	1.53
LC sheet-L	173.4	0.29	360.2	583.6	801.3	0.3170	0.54	1.53

Note: -T is the transverse scanning direction, -L is the longitudinal scanning direction, *E* is the elastic modulus of substrate and LC sheets, *μ* is Poisson’s ratio of substrate and LC sheets, *f*_y_ is the yield stress of substrate, *σ*_0.2_ is the 0.2% proof stress of LC sheets, *σ*_u_ is the ultimate stress, *σ*_neck_ is the true stress at the necking, *ε*_neck_ is the true equivalent plastic strain at the necking, *Q* is the weight parameter, *α* is the toughness parameter.

**Table 3:** Process parameters of LC.

Item	Value
Layer thickness	0.8 mm
Scanning velocity	1.2 m/min
Laser power used in this study	2200 W
Laser spot *	Rectangle, 5 mm × 2.2 mm
CO_2_ protecting gas velocity	15 Pa·L/min
Powder feeding velocity	18 g/min
Overlap ratio	60%

Laser spot *: The area on a surface illuminated by a laser.

**Table 4:** Dimensions of specimens.

Specimens	*B*_des_ (mm)	*t*_des_ (mm)	*B*_mea_ (mm)	*t*_mea_ (mm)	*d* (mm)	*S* (mm)	*t*_LC_ (mm)	Interface Slope	Scan Pattern
Int-1	40	20	39.91	19.85	-	-	-	-	-
Int-2	40	20	39.93	19.73	-	-	-	-	-
Int-3	40	20	39.99	19.62	-	-	-	-	-
Cor-1	40	20	39.89	19.65	4	10	-	1:2.5	-
S4-T	40	20	39.89	20.16	4	4	5.78	1:1	Transverse
S4-L	40	20	39.88	19.51	4	4	5.96	1:1	Longitudinal
S10-T	40	20	39.94	18.73	4	10	5.92	1:2.5	Transverse
S10-L	40	20	39.73	18.61	4	10	5.44	1:2.5	Longitudinal
S20-T	40	20	39.94	18.36	4	20	5.96	1:5	Transverse
S20-L	40	20	39.92	19.40	4	20	5.72	1:5	Longitudinal

**Table 5:** Mechanical properties of different specimens.

Specimens	*E* (GPa)	*σ*_y_ (MPa)	*σ*_u_ (MPa)	*α* (%)	*I*_y_ (%)	*I*_u_ (%)
Int-1	207.34	332.49	486.88	34.63	30.32	28.81
Int-2	200.21	340.19	483.58	32.91	33.33	27.94
Int-3	185.44	323.67	470.64	31.60	26.86	24.51
Cor-1	200.19	255.14	377.98	21.20	-	-
S4-T	170.90	319.71	481.07	28.06	25.31	27.27
S4-L	180.90	315.90	473.34	27.12	23.81	25.23
S10-T	188.02	339.48	484.74	34.26	33.06	28.24
S10-L	173.15	344.00	491.15	25.66	34.83	29.94
S20-T	197.20	355.56	498.12	27.74	39.36	31.78
S20-L	207.38	350.84	495.50	24.04	37.51	31.09

Note: *E* is the elastic modulus of specimens, *σ*_y_ is the yield stress of specimens, *σ*_u_ is the ultimate stress of specimens, *α* the is ultimate elongation of specimens.

## Data Availability

Data are contained within the article.
